# Teachers’ perceptions of artificial intelligence in curriculum integration: opportunities, concerns, and professional development needs

**DOI:** 10.3389/frai.2026.1806165

**Published:** 2026-04-07

**Authors:** Derya Kayıran, Mehtap Sönmez, Alperen Avcı, Rahma Yusuf Haji Mohamud

**Affiliations:** 1 Department of Child Development, Kahramanmaraş Sütçü İmam University, Kahramanmaraş, Türkiye; 2 Faculty of Health, Kahramanmaraş Sütçü İmam University, Kahramanmaraş, Türkiye; 3 Department of Child Development, Muş Alparslan University, Muş, Türkiye; 4 Health Services Manager, Yardimeli Specialist Hospital, Mogadishu, Somalia

**Keywords:** artificial intelligence in education, curriculum integration, professional development, qualitative research, teachers’ perceptions

## Abstract

The growing presence of artificial intelligence (AI) in educational contexts has raised important questions regarding teachers’ readiness, perceptions, and concerns about its integration into curriculum and classroom practice. This qualitative study explores in-service teachers’ perspectives on artificial intelligence in relation to instructional processes, cognitive development, psychosocial implications, and professional learning needs. The study was conducted with 30 teachers working in public schools in a metropolitan city in Türkiye. Data were collected through a semi-structured interview form consisting of eight open-ended questions and analyzed using qualitative content analysis. Coding was carried out in three stages (open, axial, and selective coding), and inter-coder reliability was calculated at 87%. The findings reveal a balanced yet cautious stance toward AI. Teachers perceived AI as a tool that can enhance instructional efficiency, support the explanation of complex content, facilitate assessment processes, and enrich students’ cognitive development. At the same time, they expressed concerns about potential risks related to emotional development, reduced interpersonal interaction, and technological dependency. Across responses, participants emphasized the necessity of structured, practice-oriented professional development to support responsible and effective AI integration. Overall, the results suggest that teachers approach AI neither with uncritical enthusiasm nor outright resistance, but with cautious openness shaped by both pedagogical considerations and ethical concerns. These findings provide context-sensitive insights that may inform teacher education and curriculum planning efforts in emerging AI-integrated educational systems.

## Highlights

- The study explores teachers’ perceptions of artificial intelligence and its integration into educational curricula through qualitative inquiry.
- Findings indicate that many teachers lack comprehensive knowledge of AI and express a strong need for professional development in this area.
- Teachers perceive AI as a tool that can enhance teaching efficiency, access to knowledge, and learning engagement.
- Concerns were raised regarding potential negative effects of AI on students’ emotional and social development.
- Results underline the necessity of integrating AI education into curricula alongside systematic teacher training programs.

## Introduction

Artificial intelligence (AI) has shifted from being a distant technological prospect to a daily reality that increasingly shapes how people learn, work, and communicate. In education, this shift has accelerated with the widespread availability of AI-powered tools that can generate text, provide feedback, personalize content, translate languages, and support administrative tasks. As a result, the question is no longer whether AI will enter schooling, but how it will be integrated in ways that are pedagogically meaningful, ethically responsible, and developmentally appropriate. International organizations have recently emphasized that AI integration requires not only infrastructure and policy, but also teacher capacity and professional judgment particularly with respect to transparency, data privacy, equity, and the prevention of over-reliance on automation (UNESCO, 2023; OECD and Education International, 2023; Miao and Holmes, 2021).

Teachers are central to this transformation because they interpret curriculum goals, decide how learning activities will be designed, and mediate the classroom consequences of new technologies. However, empirical research consistently shows a gap between the rapid diffusion of AI tools and teachers’ readiness to use them with confidence and ethical awareness. For example, teachers may recognize potential benefits such as time efficiency, differentiated instruction, and improved access to information, while simultaneously expressing concerns about students’ emotional development, dependency, academic integrity, and the erosion of human interaction (Kayıran and Avcı, 2022; Kim and Kim, 2022; Garzón et al., 2025). This duality is especially salient in early and primary education contexts where learning is strongly relational and socio-emotional development is a core educational priority.

The present study is situated within this evolving landscape and focuses on teachers’ perceptions of AI in relation to curriculum, instructional practice, and child development. This focus is timely because many education systems including Türkiye have articulated national AI strategies and policy intentions that explicitly reference the need to align education with AI-related developments (OECD, 2021). Policy documents can set direction, but the everyday feasibility and educational quality of AI integration depends on how teachers understand AI, what they consider useful or risky, and what types of support they view as necessary. In other words, teachers’ perceptions are not a “soft” variable; they are a practical determinant of whether curricular initiatives become meaningful classroom practice.

Although the literature on AI in education has grown rapidly. Particularly since 2022 important gaps remain regarding teachers’ lived perceptions and concerns, especially in contexts where AI policy ambitions coexist with uneven professional development opportunities (Wang et al., 2024; Garzón et al., 2025; Selwyn, 2019; Yıldız and Kayıran, 2023). Qualitative inquiry is well suited to illuminate these gaps because it can capture the nuanced ways teachers describe classroom realities, including the tensions between innovation and child well-being, or between efficiency and dependency. Accordingly, this study uses qualitative methods to explore teachers’ perspectives on AI, aiming to provide evidence that can inform curriculum planning, teacher professional learning, and ethical guidance for AI use in schools.

## Theoretical framework

To interpret teachers’ perceptions of AI in curriculum and classroom practice, this study draws on two complementary lines of theory: technology acceptance perspectives and teacher professional knowledge frameworks. Together, these perspectives help explain (a) why teachers may welcome or resist AI tools and (b) what kinds of knowledge are required to integrate AI ethically and effectively in teaching.

### Technology Acceptance Model (TAM): perceived usefulness and ease of use

A substantial body of research on educational technology adoption has shown that teachers’ intentions to use a technology are shaped by their beliefs about its value and usability. The Technology Acceptance Model (TAM) proposes that adoption is primarily influenced by perceived usefulness (the extent to which a teacher believes the technology will enhance teaching performance) and perceived ease of use (the extent to which a teacher believes the technology will be free of effort), which in turn shape attitudes and behavioral intentions (Davis, 1989).

Within an AI context, “usefulness” often appears in teachers’ accounts as time-saving, faster access to information, or support for planning and assessment; “ease of use” is reflected in whether teachers feel competent, confident, and supported to use AI tools without uncertainty or fear of error. Recent research applying acceptance-based approaches to AI indicates that adoption is also influenced by teacher anxiety, self-efficacy, and ethical concerns factors that can intensify when AI systems are perceived as opaque or potentially harmful (Runge et al., 2025; Ofem et al., 2025; Olaf Zawacki-Richter et al., 2019). In this study, TAM provides a useful lens to interpret why teachers may simultaneously express interest in AI integration (e.g., improved efficiency) and hesitations (e.g., fears about dependency or emotional effects).

### TPACK and the move toward AI-specific professional knowledge

While TAM helps explain “willingness” to adopt AI, it does not fully explain the quality of integration namely whether AI use aligns with learning goals, supports developmentally appropriate pedagogy, and respects ethical principles. For this reason, teacher knowledge frameworks are needed. The Technological Pedagogical Content Knowledge (TPACK) framework conceptualizes effective technology integration as the intersection of content knowledge, pedagogical knowledge, and technological knowledge, emphasizing that teachers must coordinate these domains rather than treat technology as an add-on (Koehler and Mishra, 2009).

However, scholars have increasingly argued that AI creates challenges not fully captured by generic technology-integration models because AI systems can generate content, make recommendations, and shape learning pathways in ways that raise distinct issues of transparency, bias, privacy, agency, and accountability. In response, more recent work proposes AI-extended versions of teacher knowledge frameworks. A prominent example is Intelligent-TPACK, which emphasizes teachers’ professional knowledge to integrate AI-based tools ethically and responsibly, including awareness of AI limitations and risks (Celik, 2023). Along similar lines, research on AI-TPACK has operationalized teachers’ AI-integrated competence, highlighting the importance of AI-related instructional design, ethical decision-making, and ongoing professional learning (Ning et al., 2024; Runge et al., 2025).

In this study, AI-extended TPACK perspectives help interpret teacher statements about (a) the kinds of classroom tasks where AI may be beneficial (e.g., explaining complex content, creating materials, supporting assessment) and (b) the kinds of risks teachers perceive (e.g., diminished social interaction, over-dependence, emotional impacts). These concerns map directly onto questions of pedagogical alignment and developmental appropriateness, especially when teachers describe AI as both a tool for engagement and a potential source of dependency.

### Ethics, child development, and human-centered AI in schooling

Finally, given that the present study explicitly touches on child development and psychosocial health, the framework also recognizes the growing emphasis on human-centered and ethical AI in education. International guidance highlights that schools must protect children’s rights and well-being, ensure data protection, maintain meaningful human relationships in learning, and prevent AI from becoming a substitute for genuine cognitive and socio-emotional development (UNESCO, 2023; OECD and Education International, 2023). These principles provide an interpretive anchor for teachers’ concerns about emotional development and dependency, enabling the discussion to move beyond “positive versus negative attitudes” toward a more educationally grounded understanding of what responsible AI integration could entail.

In sum, TAM informs how teachers evaluate AI’s practicality and desirability (usefulness, ease, anxiety), while AI-extended TPACK perspectives inform what teachers need to know to integrate AI responsibly (pedagogical fit, ethical judgment, developmental sensitivity). This combined framework supports a coherent interpretation of teachers’ perceptions as both adoption-related beliefs and professional-knowledge-related considerations.

### Purpose of the study and research questions

Despite the rapid expansion of artificial intelligence in educational discourse and policy, empirical evidence regarding how teachers interpret, evaluate, and emotionally position themselves toward AI remains limited, particularly within contexts where national AI strategies are developing but systematic teacher training is still emerging. While prior studies have examined technology adoption more broadly, fewer studies have qualitatively explored teachers’ lived perceptions of AI in relation to curriculum integration, child development, and professional readiness.

Moreover, much of the existing literature focuses on higher education or pre-service teachers, leaving a gap in understanding in-service teachers’ perspectives within school settings. This gap is particularly important because teachers are not merely technology users; they are pedagogical decision-makers who translate policy into practice. Their interpretations of AI whether as a supportive instructional tool, a cognitive enhancer, a potential risk to socio-emotional development, or a source of professional uncertainty directly shape how AI is enacted in classrooms.

Grounded in technology acceptance perspectives and AI-extended teacher knowledge frameworks, this study aims to explore teachers’ perceptions of artificial intelligence in relation to curriculum integration, instructional processes, and child development. Rather than assuming either optimism or resistance, the study seeks to understand the complexity of teachers’ positions, including perceived benefits, concerns, and professional development needs.

Accordingly, the study is guided by the following research questions:

1. What is teachers’ level of knowledge and awareness regarding artificial intelligence and AI-based applications?
2. How do teachers perceive the potential role of artificial intelligence in curriculum and instructional processes?
3. What benefits and concerns do teachers associate with the use of artificial intelligence in education, particularly in relation to child development and psychosocial well-being?
4. What types of professional development and institutional support do teachers perceive as necessary for effective and responsible AI integration?

By addressing these questions through qualitative inquiry, the study aims to contribute context-sensitive evidence that may inform teacher education, curriculum planning, and ethical guidance for AI integration in schooling.

## Method

### Research design

This study was designed as a qualitative descriptive case study aiming to explore in-service teachers’ perceptions of artificial intelligence within the context of curriculum integration and child development. A qualitative approach was deemed appropriate because the research sought to understand teachers’ subjective meanings, concerns, and interpretations rather than to measure predetermined variables.

More specifically, the study followed an instrumental case study design (Yin, 2018), in which a bounded group of teachers in a specific educational context was examined to generate in-depth insight into a contemporary educational issue. The case was bounded by location (a metropolitan city center in Türkiye), time (2022–2023 academic year), and participant profile (actively teaching in public schools).

Qualitative case study methodology was selected because it enables detailed exploration of real-life phenomena in context and allows participants to articulate nuanced views regarding emerging technologies in education.

#### Participants and sampling strategy

Participants were selected through convenience sampling, as the researchers accessed teachers working in schools located in the city center where the research team was based. While convenience sampling limits statistical generalization, it is commonly used in exploratory qualitative research when the aim is depth of understanding rather than representativeness.

A total of 30 in-service teachers voluntarily participated in the study. Of these, 26 were female (86.6%) and 4 were male (13.4%). Teaching experience ranged from 0 to over 20 years. Although the predominance of female participants reflects the gender distribution typical in certain teaching levels in Türkiye, this imbalance may have influenced the findings. Prior research suggests that gender may relate to differences in technology attitudes; therefore, this is acknowledged as a limitation of the study (see Table 1).

**Table 1:** Demographic characteristics of the participants (*N* = 30).

Category	Group	*n*	%
Gender	Female	26	86.7
	Male	4	13.3
Teaching Experience (years)	0–5	13	43.3
	6–10	7	23.3
	11–15	5	16.7
	16–20	3	10.0
	21 and above	2	6.7
Age	20–30	11	36.7
	31–40	10	33.3
	41–50	6	20.0
	50+	3	10.0
Education level	Associate degree	5	16.7
	Bachelor’s degree	22	73.3
	Master’s degree	3	10.0
Knowledge of artificial intelligence	Yes	24	80.0
	No idea	4	13.3
	No	2	6.7
Knowledge of AI-based applications	Yes	13	43.3
	No	17	56.7
Received AI training	Yes	3	10.0
	No	27	90.0
Need training on artificial intelligence	Yes	30	100.0
	No	0	0.0

### Data saturation

Data collection and analysis were conducted concurrently. After the 25th response, no substantially new codes emerged, and subsequent responses repeated existing categories. When thematic redundancy became evident and no new conceptual insights were identified, data collection was concluded at 30 participants. Thus, the decision to stop data collection was based on evidence of thematic saturation rather than a predetermined number.

### Transferability

Because the study was conducted within a single metropolitan context using convenience sampling, the findings cannot be generalized to all teachers in Türkiye. However, detailed demographic information and contextual description are provided to enable readers to assess the potential transferability of the findings to similar educational settings.

Ethical approval was obtained from the Mus Alparslan University Scientific Research and Publication Ethics Committee (Decision No: E-10879717-050.01.04-73838, December 2, 2022). Participation was voluntary and informed consent was obtained electronically.

### Data collection instrument

Data were collected using a semi-structured interview form consisting of eight open-ended questions. The instrument was developed following an extensive review of literature on artificial intelligence in education, teacher technology integration, and AI-related professional development needs (Appendix A).

The questions addressed four main areas:

1. Teachers’ knowledge and awareness of AI
2. Perceived areas of AI use in education
3. Perceived benefits and concerns of AI integration
4. Professional development needs related to AI

### Content validation

To ensure content validity, the draft instrument was reviewed by three experts:

- One specialist in curriculum and instruction
- One specialist in educational technology
- One qualitative research methodologist

Based on their feedback, two questions were rephrased for clarity and redundancy was reduced. A pilot test was conducted with two in-service teachers to assess comprehensibility and clarity of wording. Minor revisions were made following the pilot.

The final version of the instrument was distributed electronically to participants.

### Data collection procedure

The interview form was distributed via an online platform. Participants responded in writing to the open-ended questions. The use of written responses allowed participants to reflect on their views and respond at their convenience.

All responses were anonymized. Participants were assigned numerical codes (P1–P30) to protect confidentiality.

### Data analysis

Data were analyzed using qualitative content analysis. The analysis followed a systematic three-stage process:

Stage 1: Open Coding

All responses were read multiple times to gain familiarity with the data. Initial codes were generated inductively based on meaningful units of text.

Stage 2: Axial Coding

Related codes were grouped into subthemes based on conceptual similarity.

Stage 3: Selective Coding

Subthemes were organized into overarching themes that represented broader patterns in teachers’ perceptions.

The analysis was conducted manually using Microsoft Excel to organize codes, categories, and themes.

Inter-coder Reliability

To enhance credibility, two independent researchers reviewed the coding scheme. Inter-coder reliability was calculated using Miles and Huberman’s (1994) formula:

Reliability=Agreement/(Agreement+Disagreement)×100

The resulting agreement rate was 87%, indicating a high level of consistency between coders.

Discrepancies were discussed until consensus was reached.

### Trustworthiness and reflexivity

To ensure trustworthiness, the study followed the criteria of credibility, dependability, confirmability, and transferability.

- Credibility was strengthened through direct quotations and inter-coder agreement.
- Dependability was supported by documenting the coding process.
- Transferability was enhanced through detailed participant description.

### Researcher reflexivity

The research team consists of scholars working in education and health-related academic fields. The researchers hold professional interest in educational technologies and curriculum development. To minimize bias, reflexive awareness was maintained during coding, and multiple researchers were involved in data analysis to reduce individual interpretive influence.

### Findings

The qualitative analysis revealed four main themes and several related subthemes regarding teachers’ perceptions of artificial intelligence in curriculum integration and classroom practice. The thematic structure of the analysis is presented in Table 2.

**Table 2:** Thematic structure of teachers’ perceptions of artificial intelligence in curriculum integration.

Main theme	Subtheme	Representative codes	Example quotations (participant code)
1. Instructional and professional facilitation	Reducing instructional monotony	Engaging lessons, increased motivation, dynamic teaching	“With such applications, lessons will no longer be boring.” (P10)
	Supporting complex content	Explaining difficult topics, concretization, visualization	“AI can help explain difficult subjects more clearly.” (P12)
	Assessment and homework management	Grading efficiency, monitoring assignments, self-assessment	“Homework control can be achieved regularly through AI.” (P23)
2. Cognitive and academic development	Access to knowledge	Fast information access, efficiency, time saving	“Provides direct access to information.” (P1)
	Thinking skills enhancement	Analytical thinking, cause-effect reasoning, abstract thinking	“It can improve abstract thinking ability.” (P5)
	Creativity and divergent thinking	Imagination, innovation, productivity	“A step for divergent thinking.” (P22)
3. Psychosocial and ethical concerns	Emotional development risks	Loneliness, weakened communication	“Children may become lonely.” (P12)
	Dependency and addiction	Overuse, reduced social interaction	“It causes addiction due to overuse.” (P27)
4. Professional development needs	Institutional training	In-service courses, Ministry seminars	“The Ministry should organize applied seminars.” (P5)
	Self-directed learning	Following publications, academic reading	“Teachers should follow academic publications.” (P3)

The findings are presented under four main themes derived from the content analysis: (1) instructional and professional facilitation, (2) cognitive and academic development, (3) psychosocial and ethical concerns, and (4) professional development needs. Each theme includes related subthemes supported by representative participant quotations.

### Instructional and professional facilitation

Teachers frequently described artificial intelligence as a tool that could ease instructional processes and reduce professional workload. Their responses suggested that AI was primarily perceived as a supportive mechanism rather than a replacement for teachers.

Several participants emphasized that AI-supported applications could make lessons more engaging and dynamic. Teachers associated AI with increased student motivation and classroom stimulation.

“With such applications, lessons will no longer be boring and students’ permanent learning will be supported.” (P10)

Similarly, some teachers indicated that AI-based tools might accelerate lesson flow and provide variety in teaching methods.

“With programs based on artificial intelligence, the teacher’s job will be easier and lessons will be taught faster.” (P2)

These statements suggest that teachers perceive AI as a means of refreshing instructional practices and enhancing student engagement.

Another recurring idea concerned the potential of AI to assist in explaining abstract or difficult concepts. Teachers highlighted the usefulness of visualization and concretization, especially for younger learners.

“Artificial intelligence will provide support in explaining difficult subjects.” (P12)

“AI applications can help concretize subjects that are difficult to explain, especially during the concrete operational stage.” (P17)

Teachers thus viewed AI as a pedagogical support tool capable of strengthening comprehension, particularly in cognitively demanding topics.

AI was also associated with assessment efficiency. Participants mentioned grading, monitoring homework, and facilitating self-assessment processes.

“Regular control of homework can be achieved with artificial intelligence applications.” (P23)

“It can be an important step in students’ self- and peer-assessment processes.” (P21)

These responses reflect a perception of AI as a tool for streamlining routine instructional tasks and supporting evaluation processes.

### Cognitive and academic development

Beyond instructional facilitation, teachers frequently discussed AI in relation to students’ cognitive development and learning efficiency.

Teachers widely perceived AI as a fast and efficient source of information.

“Provides direct access to information.” (P1)

Some participants emphasized that AI could expand learning opportunities and enable students to explore topics independently.

“We can teach many concepts faster. If we use it beneficially, children can develop in many areas.” (P1)

Time efficiency and immediacy of access were repeatedly highlighted as key advantages.

Teachers also associated AI with higher-order cognitive skills such as analytical thinking and cause–effect reasoning.

“It can improve abstract thinking ability and increase imagination.” (P5)

“It can support analytical thinking and understanding cause-effect relationships.” (P30)

These views indicate that teachers do not see AI solely as a content-delivery tool but also as a potential cognitive enhancer.

Some teachers framed AI as a catalyst for creativity and innovation.

“A step for divergent thinking.” (P22)

“It enriches thinking and develops logical intelligence.” (P26)

Participants suggested that when used appropriately, AI could support flexible thinking patterns and encourage productivity.

### Psychosocial and ethical concerns

Despite the perceived benefits, teachers expressed significant concerns about the psychosocial consequences of AI use in educational settings.

A number of participants warned that excessive reliance on AI might negatively affect children’s emotional and social development.

“Children may become lonely and their emotional development can be adversely affected.” (P12)

“Communication skills may weaken.” (P15)

These concerns reflect apprehension about reduced human interaction and diminished social engagement in learning environments.

Teachers also frequently mentioned the risk of technological dependency.

“It causes addiction due to overuse and lack of social activity.” (P27)

Participants feared that uncontrolled AI use could limit children’s research motivation and reduce independent effort. These responses reveal an underlying tension between efficiency and over-reliance.

### Professional development needs

Across responses, teachers consistently expressed the need for structured professional development in artificial intelligence.

Many participants believed that national educational authorities should provide systematic training programs.

“The Ministry should organize applied seminars for teachers.” (P5)

“In-service courses should be compulsory and comprehensive.” (P7)

Teachers emphasized the need for practical, hands-on training rather than purely theoretical seminars.

In addition to institutional support, some participants highlighted personal responsibility in professional growth.

“Teachers should follow academic publications and improve themselves.” (P3)

“They should stay up to date by following current publications.” (P18)

These responses demonstrate an awareness among teachers that AI integration requires continuous learning and professional adaptation.

Overall, the findings reveal a balanced yet cautious perspective among teachers. While artificial intelligence is perceived as potentially beneficial for instructional efficiency and cognitive development, participants simultaneously express concerns regarding emotional well-being and dependency. Furthermore, teachers consistently identify professional development as a prerequisite for effective and responsible integration of AI into educational practice.

## Discussion

The present study explored in-service teachers’ perceptions of artificial intelligence (AI) in education, focusing on instructional integration, cognitive and psychosocial dimensions, and professional development needs. The findings reveal a complex landscape in which teachers recognize substantial benefits of AI for instructional processes and cognitive development, yet simultaneously express significant concerns regarding emotional development and dependency. These results both align with and extend current research on teacher perceptions of AI in educational contexts.

### Instructional and professional facilitation

Teachers in this study frequently reported that AI could facilitate instruction, reduce monotony, and streamline routine tasks, such as planning and assessment. This positive view is consistent with previous research showing teachers’ recognition of AI’s potential for personalized learning and workload reduction. For example, multiple empirical studies report that educators see AI as a tool that can automate administrative tasks, support personalized strategies, and enable efficient student performance analysis (Moura and Carvalho, 2024; Putra and Saputra, 2025).

In particular, the subthemes of reducing instructional monotony and supporting complex content reflect teachers’ interest in using AI to enhance engagement and comprehension. This aligns with findings by Mardhiyya et al. (2025), who reported that teachers viewed AI as helpful for generating ideas, tailoring content, and offering timely feedback, thereby contributing to lesson planning and strategy development. Similarly, high levels of perceived usefulness and perceived ease of use, concepts central to technology acceptance theories such as the Technology Acceptance Model (TAM), have been documented as positive predictors of teachers’ AI adoption (Yim and Wegerif, 2024).

Taken together, these studies suggest that when teachers perceive clear instructional benefits and manageable integration processes, they are more likely to view AI as an asset rather than a threat.

### Cognitive and academic development

Teachers in our study also associated AI with improved access to knowledge and cognitive advancement, encompassing analytical thinking and creativity. This is in line with research evidencing teachers’ beliefs that AI can support differentiated instruction and enrich learning experiences, particularly when it facilitates exploration and individualized support (Çetingüney and Büyük, 2025; Uygun, 2024).

For instance, Cruz et al.’s (2024) investigation of STEAM teachers found that participants perceived AI as valuable for designing pedagogical experiences that enhance student engagement and innovation. These perceptions mirror the present study’s findings, where teachers described AI as a tool with pedagogical flexibility and potential to support higher-order thinking.

However, it is important to note that cognitive benefits are not uniformly perceived. Some literature suggests that teachers’ AI self-efficacy and prior experience significantly influence their attitudes toward using AI tools for learning support; higher self-efficacy often correlates with more positive perceptions (Attitudes, perceptions and AI self-efficacy in K-12 education, 2024). This complexity underscores the need to consider individual differences in professional knowledge and confidence when interpreting teachers’ cognitive appraisals of AI.

### Psychosocial and ethical concerns

Despite acknowledging benefits, participants expressed concerns about emotional development risks and technological dependency. These concerns resonate with broader discussions in the international literature regarding AI’s ethical implications in education. For example, ethical and social considerations, including privacy, equity, and potential negative effects on communication skills, have been repeatedly highlighted as critical areas requiring attention (Yurtsever and Yurtsever, 2025; Moura and Carvalho, 2024).

Similarly, in recent findings from Indonesia and other regions, teachers highlighted limited digital literacy and inadequate training as barriers to effective AI integration, in addition to concerns about over-reliance and potential negative impacts on students’ independent skills (Putra and Saputra, 2025; Shah et al., 2024). These apprehensions align with international policy discussions emphasizing that AI integration must be balanced with safeguards to protect interpersonal engagement and socio-emotional development in schools. Specifically, several international reports stress the importance of human-centered AI use that does not diminish students’ autonomy, creativity, or social interaction (UNESCO, 2023; OECD and Education International, 2023).

Taken together, these perspectives suggest that teachers’ concerns in the present study are not isolated opinions but reflect broader, well-documented tensions in educational AI discourse.

### Professional development and institutional support

A consistent theme across teacher responses was the need for structured professional development. Teachers emphasized both institutional training and self-directed learning as prerequisites for responsible AI integration. This finding aligns with systematic reviews showing a gap between policy aspirations and teacher readiness; many educators report insufficient formal training opportunities and express the need for targeted, practical support (Tan et al., 2024; Prilop et al., 2025).

Professional development is fundamental to enhancing both AI literacy and pedagogical capacity, thus supporting not only adoption but also critical engagement with AI-based tools. Indeed, research indicates that without intentional capacity-building mechanisms, teachers’ positive perceptions may not translate into effective classroom practices (Prilop et al., 2025; Celik et al., 2022).

The convergence of findings across different contexts suggests that teachers generally hold balanced perspectives on AI: they recognize potential instructional and cognitive benefits, but these are tempered by concerns about psychosocial effects and dependency. Importantly, this balance corresponds with contemporary literature emphasizing the need for a contextual, teacher-centered approach to AI implementation one that acknowledges both potential and risk.

From a practical standpoint, this implies that curriculum developers and policymakers should prioritize:

1. Tailored professional development that builds AI literacy and pedagogical integration skills.
2. Ethical guidelines and frameworks that address privacy, equity, and socio-emotional considerations.
3. Collaborative platforms where teachers can share experiences and strategies for responsible AI use.

In doing so, the field can move beyond polarized narratives about AI as either threat or panacea, toward nuanced strategies that empower teachers as informed agents in shaping AI-mediated educational futures.

## Conclusion

The rapid expansion of artificial intelligence in educational discourse has created both opportunity and uncertainty for teachers. This study sought to explore how in-service teachers perceive artificial intelligence in relation to curriculum integration, instructional practice, and child development. The findings reveal a nuanced and balanced perspective rather than a polarized stance.

Teachers in this study recognized the instructional and cognitive potential of AI, particularly in terms of increasing efficiency, facilitating access to knowledge, supporting complex content explanation, and enriching classroom engagement. At the same time, they expressed reservations regarding the possible psychosocial consequences of excessive AI use, including emotional detachment, weakened interpersonal communication, and technological dependency. These concerns suggest that teachers do not approach AI as a purely technical tool but as a phenomenon that carries pedagogical and developmental implications.

A consistent outcome across participants was the perceived need for structured and practice-oriented professional development. Teachers indicated that without adequate training, ethical guidance, and institutional support, meaningful integration of AI into educational settings would remain limited. This highlights the importance of aligning technological innovation with teacher preparedness and reflective pedagogical judgment.

Importantly, the findings should be interpreted within the study’s contextual boundaries. The research was conducted with 30 teachers from a single metropolitan area using convenience sampling. The predominance of female participants may also have influenced the perspectives reported. Therefore, the results are not intended to be generalized statistically but rather to offer context-sensitive insight into how teachers interpret AI at the classroom level.

Future research could expand this inquiry by including more diverse geographical regions, balanced gender representation, and comparative analyses across educational levels. Additionally, longitudinal studies examining how teachers’ perceptions evolve as AI tools become more embedded in daily practice would contribute valuable understanding to the field.

In conclusion, the study suggests that teachers approach artificial intelligence neither with uncritical optimism nor outright resistance. Instead, their perspectives reflect a cautious openness—recognizing potential instructional benefits while remaining attentive to ethical and developmental considerations. Supporting teachers through structured professional learning and reflective dialogue may therefore be a key step toward responsible and contextually appropriate AI integration in education.

### Limitations

This study should be interpreted in light of several limitations. First, the research was conducted with 30 in-service teachers from a single metropolitan area in Türkiye using convenience sampling. Although qualitative research does not aim for statistical generalization, the contextual nature of the sample limits the transferability of findings to different geographical regions or educational systems. Second, the predominance of female participants (86.6%) may have influenced the perspectives reported. While this distribution reflects the gender composition of certain teaching contexts, it may also shape attitudes toward technology integration. Future studies with more balanced gender representation would allow for deeper comparative analysis. Third, data were collected through written responses to a semi-structured interview form administered electronically. Although this method enabled reflective responses, it may have limited opportunities for probing and follow-up clarification that are possible in face-to-face interviews. Fourth, the study captures teachers’ perceptions at a particular moment in time. Given the rapidly evolving nature of artificial intelligence technologies, teachers’ attitudes and competencies may shift as AI becomes more embedded in everyday educational practice. Despite these limitations, the study provides context-sensitive qualitative insights into how teachers interpret the opportunities and challenges of AI integration at the curriculum level.

## Data Availability

The raw data supporting the conclusions of this article will be made available by the authors, without undue reservation.

## Appendix A

### Semi-structured interview questions

The semi-structured interview protocol consisted of eight open-ended questions designed to explore teachers’ perceptions of artificial intelligence in educational contexts. The questions were reviewed by experts in curriculum and educational technology to ensure content relevance and clarity.

The following semi-structured interview questions were developed to explore teachers’ perceptions of artificial intelligence in relation to curriculum integration, instructional practices, and its potential effects on students’ development. The questions were reviewed by experts in curriculum and instruction and educational technology before implementation.

1. *How do you define artificial intelligence in the context of education?*

What does the concept of artificial intelligence mean to you as a teacher?

1. *What kinds of roles do you think artificial intelligence can play in teaching and learning processes?*

In which areas of the classroom or curriculum do you think AI tools could be useful?

1. *In your opinion, how might artificial intelligence contribute to students’ learning and cognitive development?*

For example, could it support skills such as problem solving, analytical thinking, or creativity?

1. *Do you think artificial intelligence can help teachers in instructional planning, assessment, or classroom management?*

If yes, in what ways?

1. *What concerns or risks do you think may arise from the use of artificial intelligence in educational settings?*

For instance, could it affect students’ social interaction, motivation, or emotional development?

1. *How prepared do you feel as a teacher to use artificial intelligence tools in your teaching practice?*

What kind of knowledge or competencies do you think teachers need?

1. *What type of professional development or institutional support would help teachers integrate artificial intelligence into the curriculum effectively?*
2. *Overall, how do you evaluate the future role of artificial intelligence in education and curriculum development?*
